# Social Overview of Smartphone Use by Teenagers

**DOI:** 10.3390/ijerph192215068

**Published:** 2022-11-16

**Authors:** María-Carmen Ricoy, Sara Martínez-Carrera, Isabel Martínez-Carrera

**Affiliations:** Department of Didactics, School Organization and Research Methods, Faculty of Education and Social Work, Universidade de Vigo, 32004 Ourense, Spain

**Keywords:** adolescence, digital press, education prevention, emotions, ICTs, smartphone, socialization

## Abstract

Information and Communication Technologies have led to a new way of life and, in particular, of socialization. The objective of this study is to analyse the image social media disseminate of news taken from digital newspapers, based on the opportunities and drawbacks attributed to smartphone use by teenagers. An essentially qualitative methodology was used, on a sample of 1704 news items published in digital newspapers. The results and conclusions show that smartphone use by teenagers improves development of their digital competence, presents new academic opportunities (through gamification or mobile learning) and provides them with digital tools for school and leisure. The widespread drawbacks reflect the effects of the device on the deterioration of health (dependence, stress, psychosocial problems) and emotions, thereby succinctly affecting academic performance. A noticeable increase of positive news about smartphones was published in the major newspapers in December, while that on its negative effects, in September.

## 1. Introduction

The bloom of Information and Communication Technologies (ICTs) has created a new socialization structure by changing the way people communicate and live. ICTs play an important role in the daily lives of citizens because they are permanently present as part of the immersive digital culture. However, they can generate an undesirable dependence [1]. In like manner, digital development is an essential platform for digital journalism which has acquired great prominence due, among other aspects, to its immediacy and impact [2]. The exclusivity of the analogue press is now extinct, after ceding its space to the more versatile digital press with a very wide and ambitious outreach.

The media, including newspapers now present a continuous flow of information and generally provide unhindered news and events with a multitude of approaches [3]. The digital press, and in particular, daily newspapers, offer a great deal of information that has a huge influence on citizens. The news is quite varied, albeit of events, situations or phenomena that can generate a greater impact on people, as well as social or socio-political controversy.

Teenagers’ use of ICTs is worrying, especially with mobile devices such as smartphones. Possible dysfunctional uses include phubbing, fomo, selfiphobia, nomophobia, vibranxiety and sexting [4,5,6]. Digital newspapers echo this psycho-social concern and the multiple problems generated by the device, thus reflecting the reality.

Daily newspapers provide information that can influence citizenry and therefore, credibility should be one of its main objectives. The press generates social interest because all of its formats and media, in some way or another not only reflect everyday life and facilitate learning, but also portrays reality, albeit from different angles. The Internet, together with education professionals and the family, should offer teenagers the tools needed to learn, think and question information. Digital literacy is a learning process that requires training and dedication [7]. Reading on-line news in a critical and constructive manner is essential for an informed and engaged citizenry, but they need to be taught and trained.

Digital press news continues to rise in parallel with the growth of mobile devices, since the technology is used by a large number of people. The vast majority opt for digitization in their social relations [8]. Therefore, educational professionals should contribute to or reinforce media literacy in youngsters, in order to train citizens to have a reflective and critical viewpoint. ICTs have changed the way content is consumed and provide diversified access to information. However, digital media content is now more intermingled than ever, making it difficult to read, analyse and understand. Therefore, teaching and learning to read on-line news in a critical and constructive manner is essential for establishing an informed and engaged citizenry [9]. More and more people are using digital and mobile devices to read newspapers, social media or to play games, and, in general, to indiscriminately surf the Internet.

The 2021 Report on Digital News Users in Spain [10], published by the Universities of Navarra and Oxford, indicates that the mobile phone is the most used device by Internet users to seek information: 9 out of 10 (90%) use it regularly for some purpose and 8 out of 10 (78%) for news. This is five points higher than in 2020 and 11 points higher than in 2019 (67%). On the other hand, according to the latest Annual Report of the National Observatory of Telecommunications and the Information Society [11], this is the first time ever that there are more mobile lines than inhabitants in developing countries. Telephone land lines are widespread worldwide, but are in a clear downward trend. In the last five years, the number of land lines per 100 inhabitants decreased by 4.6 in developed countries, while the decrease was 2.5 lines per 100 inhabitants in developing countries. In the aforementioned 2021 document on Digital News Users in Spain, it is also stated that the mobile telecommunications are becoming increasingly popular in different countries. They are growing the fastest in developing countries, from 99.4 to 103.8 lines per 100 inhabitants. It should be noted that there are 128.9 mobile phone lines per 100 inhabitants in developed countries. This is because the same user can have a private and another professional mobile phone line. Another important point is that, over the past five years, the Asia-Pacific and Africa regions have led the growth of mobile phone lines. The increase was smaller in the Americas and the Commonwealth of Independent States, and a decrease is observed in Europe and the Arab States. On the other hand, smartphone expansion is striking; for example, in Mexico, where it has a penetration rate of 45.6% with 59,597,000 users, and in Colombia 39.8% with 19,669,000 users [12].

According to data from the Association for Media Research [13], 14% of Spanish households own exclusively cell phones, abandoning fixed telephony. Smartphone availability in the population of Spanish children (aged 10–15 years) is very high. About 70% of teenagers have the device and acquisition increases with age, reaching 99.1%, in those aged 16+ years [14,15]. Smartphones are the most widely used digital mobile devices among teenagers and youngsters in Spain. However, research in other countries shows that smartphone use as a digital consumer device is not as high.

Mobile phones are used by teenagers practically all day long, both for leisure and communication purposes [16,17,18]. They therefore need to be protected at a key moment in their psychological, social and emotional development. In this sense, it is important to highlight the importance of improving and reinforcing their emotional development, with a view to strengthening well-being and helping them cope and manage affections and the multiple situations they face [19]. At a vulnerable stage such as at adolescence, we need to be careful about how they use mobile phones and interact with ICTs [20]. Education professionals and family should guide teenagers in using smartphones correctly and protecting themselves from the dangers of the Internet. Also noteworthy, is that this vital stage, is the right time to build healthy habits that can last a lifetime.

## 2. Justification and Purpose of the Study

The relevance of the smartphones and the tablets is massive. This has made them quite popular, particularly among teenagers and youth. However, we need to analyse the impact of smartphone use at an early age, in order to assess possible consequences, especially if they can be of a very problematic or irreversible nature.

This investigation is the first of its kind. The study presents the social panorama that Spanish digital newspapers spread about smartphones use among teenagers and the temporal affluence with which news are published. This analysis is a starting point for planning psycho-socio-educational prevention strategies to help raise awareness to society, educators and the family environment about the benefits and risks of smartphone use by adolescents.

The aim of this study is to understand the image offered by social media, including digital newspapers, on smartphones use by teenagers. This study was guided by the following research questions (RQ):

− RQ1: What are the benefits attributed by digital newspapers to teenagers that use smartphones?
− RQ2: What are the drawbacks that digital newspapers spread about smartphone use by teenagers?
− RQ3: What is the temporal distribution of newspaper news items on smartphone use by teenagers?

## 3. Materials and Methods

The present work follows an essentially qualitative methodology, framed within the narrative approach, based on a documentary study [21]. In fact, the study focuses on the analysis of news items from digital newspapers.

### 3.1. Procedure and Sampling

An exploratory search on the Internet was initially performed relating to smartphone use by teenagers, in order to select the study sample. The sample was subsequently determined using chosen criteria. The criteria chosen to delimit this digital newspapers’ sample, taking into account items published in the last four years (1 January 2018 to 31 December 2021), is as follows:

1. Spanish context;
2. Availability in the Digital Newspaper Library of the National Library of Spain (Ministry of Culture and Sports);
3. Higher visibility index in Sistrix;
4. Best score in Comscore data from 2018 to 2021;
5. Media popularity among citizenry;
6. Inclusion of media with diverse ideologies, to allow for greater plurality.

**Figure 1:**
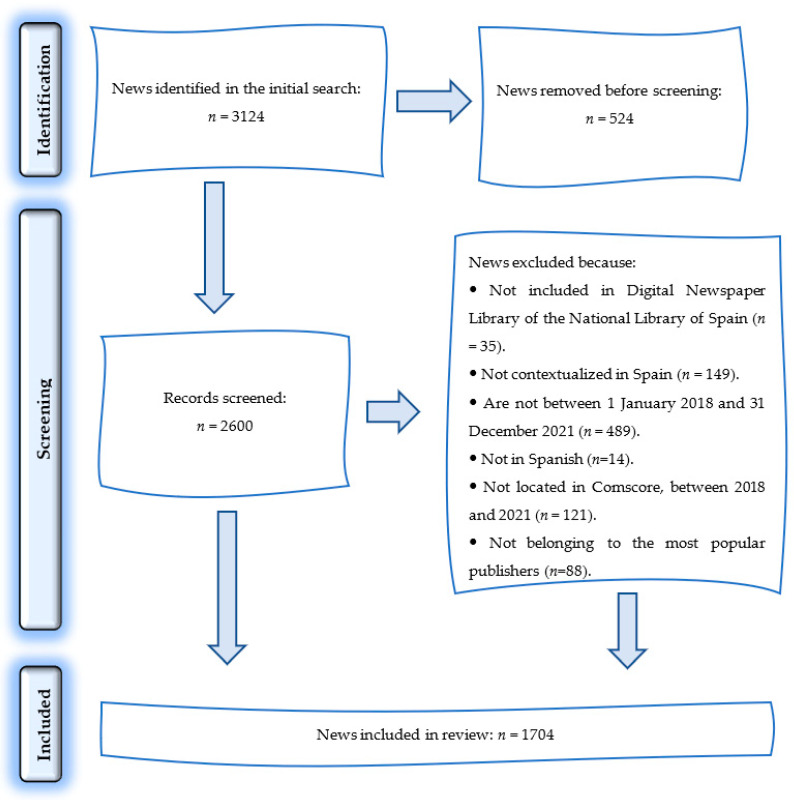
News selection procedure for delimiting the sample.

The PRISMA 2020 statement guidelines were also used for searching and selecting news from digital newspapers [22] (Figure 1).

Insofar as the theme for news items selection from digital newspapers is concerned, the ones linked directly to smartphone use by teenagers have been taken into account. The Google News tool and news clippings from digital newspapers have been very useful for this purpose.

The final delimitation of the news sample was done after taking into account their link with the research topic and the following descriptors: “smartphone, teenagers”; “smartphone, teens”; “smartphone, minors”; “mobile phone, teens”. The final sample was configured using newspaper search engines, of the type Google News, that present three blocks of information (for each item from the lists of results), with the following data: title of news item, source, and a summary of the same. This facilitated final selection of the news items and the full text. The final sample included a total of 53 newspapers, with 1704 news items relating to the study topic.

### 3.2. Data Analysis

The starting point for addressing content analysis was focused on the overall objective and the three research questions. Advice from specialists was sought for the construction of codes and executed by pairs of researchers. They firstly reviewed the scientific literature and, then consulted four experts from Spanish universities to increase level of objectivity, adequacy and credibility of the final codification.

The inductive procedure was followed when categorizing data for first-level coding, based on the three research questions (Table 1). The deductive process was combined with the inductive process for 2nd and 3rd level coding. A deductive process was followed for 2nd level coding associated with smartphone use benefits, drawbacks, and temporal distribution. However, since no taxonomy was identified in the theoretical corpus to delimit the codes for 3rd level sub-categorization, an inductive process was chosen, which provided an unpublished codification [23].

Content analysis was done by using Excel (version 2016) and the Analysis of Qualitative Data (AQUAD) program, version 7. Information codification involved combining the autonomous work of the researchers (codification of raw data) with that of collaborative work (to unify codification criteria and compare and resolve codification interpretation differences). Data transfer from AQUAD to Excel facilitated an organized and substantial presentation of the results, besides providing the encoding frequency count. However, this being qualitative research, results analysis is not focussed on a purely quantitative enhancement, that is, irrespective of whether, for example, it is useful to ascertain the degree of prevalence of the type of news disseminated.

## 4. Results

**Figure 2:**
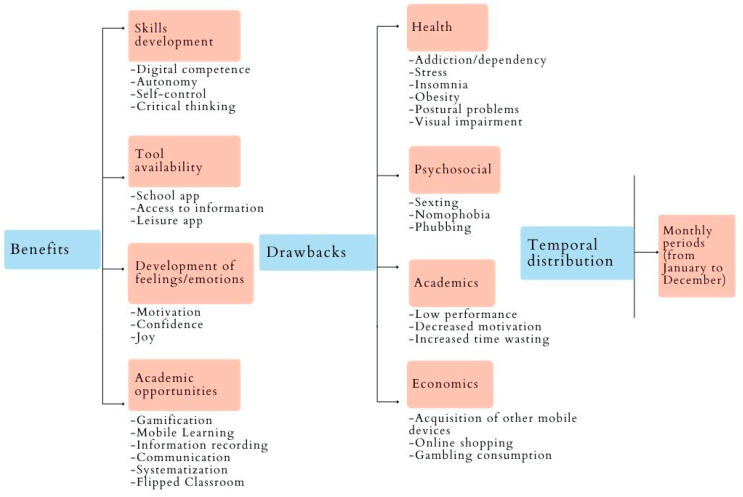
Relationship between the obtained categorisation.

Figure 2 provides an overview of the relationship between the main categories and the encoding obtained from the 2nd and 3rd levels. The different sub-headings delve in-depth into the results based on the three research questions. The results presented are accompanied by some illustrative extract to aid in their interpretation and comprehension.

### 4.1. Benefits Attributed to Smartphone Use in News (RQ1)

Multiple newspaper news items highlight development of competences by teenagers through smartphone use. In the case of digital competence (958/1704), the news mentions a greater development of knowledge and skills by them, as a result of using smartphones. Also attributed among others, is the improvement and strengthening of personal autonomy (449/1704), based on their ability to self-sufficiently accomplish tasks with smartphones (Table 2).

Another parallel benefit of smartphone use by teenagers, reflected in digital newspapers, is the one associated with self-control (147/1704). Also emphasized is the role smartphones play in the emotional regulation of teenagers, both in the use of the device and in its extrapolation to other situations.

Newspapers also mention them leveraging the tools provided by smartphones. In this sense, they mention some Apps used for school content (Kahoot!, Socrative, Duolingo), for leisure (Minecraft Earth, Pokemon Go, Jurassic World Alive), and for access to information or diverse documentation (Edmodo, Trello, Additio App). Several news items (698/1704) state that teachers have become facilitators and managers of academic knowledge, via digital tools (Apps). Several news items (411/1704) present the mobile phone as a facilitator of access to information.

Newspapers highlight the great amount of instant information available to teenagers on their mobile phones. Some news reports indicate that this information is related to recreational and school activities. Newspapers such as: *EL CORREO, elDiario.es*, and *ABC*, sustain that some Apps (Among Us, Candy Crush Saga, Angry Birds Blast), despite essentially being leisure applications, provide important benefits to the lives of teenagers. Among these, they mention information on emergent issues. They likewise highlight that the Leisure Apps (398/1704) are the ones most demanded by students and are useful to them in their spare time.

Different news items mention that smartphones are used by teenagers for the performance of daily activities (interaction with their peer group, educational activities, leisure activities, etc.), and that they play a role in the enhancement of their feelings and emotions. The device is likewise associated with the development of motivation (413/1704), given that smartphones offer teenagers the possibility of achieving objectives. In this respect, the following extract is provided:

The use of mobile devices motivates and keeps students focused, so that content is assimilated more quickly (…), helps them to become more decisive and self-reliant, learn to work as a team, develop more critical thinking, increase motivation and it also makes teaching more flexible, since the support materials provided can be adapted to the needs of every student (*20minutos*, 17 August 2021; news item no. 878) (accessed on 23 October 2021).

Another aspect mentioned by newspapers (216/1704) is greater self-confidence generation in teenagers by smartphones. In this sense, they state that the use of the device, during leisure time, strengthens their sense of well-being, fundamentally through communication with their peers.

Academic opportunities seized by teenagers using smartphones are also covered in the different newspapers (309/1704), and are mainly related to the use of innovative learning strategies.

On another note, newspapers highlight the educational impact of gamification (309/1704). In this sense, it is indicated that students use the device to manage multiple resources and digital tools for academic purposes. Moreover, digital gamification is presented as an incentive for teachers since it contributes to student motivation. In accordance with certain educational strategies, teachers personalize different academic activities and content, to address students’ needs on an individual basis. Some press reports (267/1704) reveal that teenagers increasingly use smartphones for mobile learning. The following excerpt illustrates the case:

Experts recommend not to associate tablets and mobile phones with leisure, or books and notebooks with study, because in the 21st century, gamification has arrived to not only improve and make teaching more efficient to benefit students, but also to boost their development and, in short, their experience and progress at school (*europapress*, 08 September 2021; news item no. 1128) (accessed on 23 October 2021).

Some newspaper reports (218/1704) show that teachers can keep a track of students’ academic activity via smartphones. As an example, they mention correction of exercises or academic tasks and the instant feedback possibility (186/1704). Other news items published on the academic field are associated with flipped classrooms (103/1704). In this sense, they highlight students’ interest in smartphones for performance of tasks in digital format.

### 4.2. Drawbacks Regarding Smartphones (RQ2)

Newspaper reports highlight a number of concerns associated with smartphone use by teenagers. They emphasize the negative impact the device has on their health by generating dependence/addiction behaviours, thus causing stress, insomnia, obesity, as well as postural and visual problems (Table 3).

Multiple newspaper reports (386/1704) alert the public to the dependence or addiction behaviours that excessive use of smartphones can generate in teenagers; and indicate that it tends to cause low academic performance and deterioration of their interpersonal relationships. Also mentioned, is that children feel anxiety when they cannot use the device. The publication of these alarming manifestations by newspapers is primarily aimed at alerting families and educational professionals.

Some newspaper reports (114/1704) sustain that misuse of smartphones can seriously impair sleep and cause significant deterioration in teenagers; which in turn, could lead to stress (216/1704) and obesity (92/1704). The following excerpt explains the same:

(…) of the teenagers spent more than five hours a day using those devices. The chances that these kids would drink sugary drinks, not do physical activity, or not get enough hours of sleep could be up to twice as much as their peers who used such technologies for less time. There is yet another key element: Stress. It has been proven that an intense use of mobile phones increases levels of cortisol, the so-called stress hormone, that makes us react in fight or flee situations (*elDiario.es*, 15 September 2019; news no 1007) (accessed on 23 March 2021).

Other negative consequences reflected in different newspapers are the ones produced by sexting (284/1704), nomophobia (252/1704) and phubbing (102/1704). Insofar as sexting is concerned, the press reveals that public exposure of teenagers, to sexual content images or videos through smartphones, can lead to feelings of humiliation, mistrust, loss of self-esteem and social isolation. Newspapers warn that this practice, although initially voluntary, could eventually harm privacy.

Some of them indicate that teenagers have access to smartphones at an early age, which in turn gives them access to all kinds of content with few or no security measures. As indicated above, another of the dangers that the newspapers highlight is linked to phubbing (102/1704). Some published news (252/1704) indicates that teenagers are afraid of being without their phone or not having signal coverage (nomophobia). In this respect, the following extract is provided:

The emergence of mobile phones among the youngest has created new phobias related to dependence on the device. Among these new forms of addiction, there is one very subtle one that undermines our daily lives and becomes an integral part of all our actions. It is called nomophobia. (…) or phobia that arises when unable to access a working mobile phone. (…), state of distress that pervades the individual upon the idea of losing his/her smartphone, running out of battery, continuously checking the phone to check for notifications, keeping it on 24 h a day, taking it to bed, using it at the table or at work (*salamanca24h.com, 29 September 2019; news item no. 1026*) (accessed on 12 November 2021).

At the academic level, the newspapers reflect multiple disadvantages derived from smartphone use by teenagers. For example, low academic performance is mentioned in different news items (268/1704).

Our study revealed the possible existence of economic problems generated by smartphone use in the teenagers’ group. In this sense, some news items (98/1704) reveal their craving to acquire the latest devices. The launch of new models into the market arouses great interest in them, which increasingly causes a greater expense to families.

A small news group (57/1704) reveals that gambling via smartphones, is yet another major danger. Although under 18 years of age, they are able to access some Apps destined for adults by committing identity fraud. Some leisure games chosen are poker, sports forecasts, etc. The following is an excerpt:

In particular, 18.3% of boys, compared to 2.2% of girls, play on-line games as a form of leisure and social relationships. The ones already addicted (1.22%) are aware that they must stop playing, but acknowledge that they need to bet more and more to achieve the desired effects, which causes them financial losses. They also admit that they sometimes have lied about their involvement in games and that they obtain financing from others, which is the only way to pay off their debts. In this sense, they warn that in the case of on-line betting by teenagers, the conversion from social player to a player with problems happens “quite fast” and “severely affects all teenager phases since there are no protection measures in place” and because gambling is “easily available and accessible” (*HERALDO*, 11 June 2020; news item no. 658) (accessed on 24 November 2021).

### 4.3. Temporal Distribution of News (RQ3)

Three of the newspapers analysed have published a striking number of news items on smartphone use by teenagers (*ABC*, 201/1704; *europa press*, 162/1704; and *La Voz de Galicia*, 134/1704). However, others that published with a scant frequency on the subject were: *EL DIARIO VASCO, Noticias de Gipúzkoa* and *La Voz De Asturias* (1/1704), respectively (during November, August and May) (Table 4).

The number of news items per month oscillated between 49 (in July) and 340 (in September), the average being 142 in the period analysed (1 January 2018 to 31 December 2021).

News items attributing benefits to smartphone use by teenagers were concentrated in December of the study period (568/1704), wherein some highlighted the supply of digital tools (192/821). Fewer news items associated with feelings or emotions (motivation, confidence and joy) and academic opportunities facilitated by smartphones to teenagers were published in January (Table 5).

Newspapers with the most news on smartphone use benefiting teenagers, include the following: *ABC, europa press* and *20minutos*; while those that published least on this topic are the ones with a small print run: *Lanza, La Voz de Asturias* and *Noticias de Gipúzkoa*.

September is the month with the most news on drawbacks of smartphone use by teenagers (324/1704). In this sense, many news items (973/1704) publish setbacks that smartphone use can cause to health.

There was little news published on problems related to the academic field from January to May. Likewise, hardly any news was published in January, March, April, August and November, about the possible economic consequences of smartphone use on teenagers (Table 6).

Among newspapers that published most on the drawbacks are major ones such as *europa press, elDiario.es and 20minutos;* while the ones that reported the least were minor local newspapers with a smaller print run (*alerta digital*, *EL DIARIO VASCO, DIARIO DE NAVARRA*).

## 5. Discussion

The opportunities attributed by newspaper media to smartphone use by teenagers are essentially linked to the development of digital competences, which would ensure safer use of ICTs in general and smartphones in particular. However, the so-called digital native young generations, need to learn to integrate the development of digital competence into the different areas of their lives [25]. To this end, and in the case of teenagers, the family, the school and the social environment have an essential role to play in the challenges associated with digitization [26].

From the present study it becomes clear that another of the benefits highlighted by newspapers, on smartphone use by teenagers, is the availability of digital tools (school applications, leisure applications and access to information or documentation). In this regard [27] indicate that many leisure Apps have valuable elements that encourage peer-to-peer collaboration and interaction.

The newspapers reflect that teenagers regularly use smartphones in leisure activities and in informal communication. On the other hand, it should be noted that the academic opportunities facilitated by smartphones to teenagers are rather few. This limited dissemination of news associated with academic tasks could be due to the public doubting the credibility of the device for pedagogical purposes, although some studies indicate that boys frequently and repeatedly use smartphones for school tasks [28]. Moreover, and within a context of continuous technological development and transformation, some countries are already using smartphones with teenagers, to address emerging or innovative learning strategies [29,30]. An example of this is a pedagogical strategy called gamification that relies on mobile devices such as smartphones. Some reasons that may have contributed to the expansion of gamification in schools lie in the technological profile of teenagers [31]. Moreover, schools resorted to remote-learning, and teenagers hadto rely on various technologies, for example during the pandemic [32].

This study of newspaper news items indicates that the promotion of positive feelings and emotions (motivation, confidence and joy) in teenagers, produced by smartphone use, reinforces their motivation, confidence and enthusiasm. Ref. [17] mentions the association of emotions (such as gratitude or interest) with mobile phone use.

Newspapers indicate that the drawbacks from smartphone use by teenagers are: health deterioration, psychosocial problems, and difficulties in the academic and economic contexts. They highlight the generation of dependency or addiction behaviours in teenagers. In this sense, ref. [33] also sustain that youngsters constantly use this mobile device.

The present study shows that smartphone use by teenagers has created new phobias such as phubbing or nomophobia. These issues can jeopardise teenagers’ lives by becoming part of their daily habits. Newspapers warn that the main concerns of families are that their children may misuse their smartphones. Of special concern to them is the development of sexting practices [34,35]. Given that sexting involves intimate content, such diffusion would imply a loss of privacy and cause grievance to the victim’s reputation, deterioration of his/her public image, loss of self-esteem, etc. Some studies have also found these adverse effects [36,37,38].

The news about smartphone use by teenagers succinctly reflects the poor school performance it causes and the consequent deterioration of academic qualifications. Although scientific literature echoes the drawbacks triggered by this device, it also attributes great educational potential by highlighting its positive effects on learning [39,40].

The main issues reported by newspapers about smartphone use by teenagers are related to the needs of their daily lives and the influence of the environment. This, along with new habits acquired, marks a turning point in the use initially attributed to smartphones [41]. For example, teens use them for on-line shopping and gambling. These games are pleasant and entertaining, but can trigger frustration and great concern or family problems.

The distribution of news published in the newspapers over the past 4 years is quite irregular. December was the month with the largest number of news items published on benefits of smartphone use in teenagers that emphasized the interest for diverse digital tools. On the other hand, September was the month in which there was abundant news on the drawbacks of the device. This may be conditioned by the fact that the academic year begins in September in many countries. Thus, the news warns general readers and, particularly families and professionals in the psycho-socio-educational field, about the possible problems generated by smartphone use in minors [42]. Such news furthermore contributes to encouraging reflection and debate in the public sphere, and recommends incorporation of prevention measures and alerting those affected.

The competent administration, society, educators and the family environment must be aware of the risks of smartphone use by teenagers in order to act accordingly [43]. Therefore, psycho-socio-educational prevention actions from the school environment must offer targeted protection to minors. Hence, the family must be actively involved as a fundamental and necessary support unit [44]. Multidisciplinary intervention measures are furthermore recommended to encourage responsible use of mobile devices and to strengthen their digital, emotional and psychosocial skills [45]. Clear and effective standards must be established to guarantee proper use of ICTs in the family environment and in schools [46,47]. This requires agreement or consensus to be reached on usage rules and times by/with teenagers.

## 6. Conclusions

The present study shows that smartphone use by teenagers is mainly associated with beneficial practice patterns (mainly linked to the development of digital competence, availability of resources for leisure, promotion of feelings or emotions, and motivation). However, such use is linked to a lesser extent with negative effects (health deterioration), psychosocial problems (sexting, nomophobia and phubbing), academic problems (poor school performance), and economic problems (on-line shopping and gambling).

There is a marked trend in the widely read newspapers of publishing positive news on smartphone use by teenagers in December; but most of them published the negative effects of the device in September.

## 7. Study Limitations and Future Prospects

This study offers a view of reality after analysing news published only in the main Spanish digital newspapers. Future research on the use of smartphones by teenagers should therefore contain a geographically extended sample and include newspaper news items from different countries. Probably, another limitation of this study derives from the ideological/political, cultural and economic biases that may be present in the digital newspapers under analysis. It would be interesting to include the perspective of teenagers, education professionals and the family to contrast the results.

Future studies should analyse the view of social networks on smartphone use by teenagers; as well as delve in-depth into the family problems arising as a result of such use by teenagers.

## Figures and Tables

**Table 1:** Main category definitions.

Category Name (1st Nivel)	Definition
Benefits of smartphone use (RQ1)	They are identified from the news that refer to skills development, feelings/emotions promotion, tools and academic opportunities derived from the use of smartphones by adolescents [18].
Drawbacks related with smartphone use (RQ2)	They are determined from news that reflect negative, harmful or inconvenient circumstances/situations generated or triggered by the use of smartphones in adolescents: health, economic, psychosocial and academic problems [24].
Time distribution of the news about smartphone (RQ3)	It is associated with the temporal distribution of the news, in the respective months (gregorian calendar).

**Table 2:** Interest that the news gives to the use of smartphone.

Categorization (1st Level): Benefits		Σ
2nd Level	3rd Level	f (%) *
Developed skills	Digital competence	958 (56.22)
	Autonomy	449 (26.34)
	Self-control	147 (8.62)
	Critical thinking	138 (8.09)
Tool availability	School app	698 (40.96)
	Access to information	411 (24.11)
	Leisure app	398 (23.35)
Developed feelings/emotions	Motivation	413 (24.23)
	Confidence	216 (12.67)
	Joy	120 (7.04)
Academic opportunities	Gamification	309 (18.13)
	Mobile Learning	267 (15.66)
	Information recording	218 (12.79)
	Communication	186 (10.91)
	Systematization	154 (9.03)
	Flipped Classroom	103 (6.04)
		Total: 1704

* Legend: Σ = summation; f = Absolute frequency; % = Relative frequency.

**Table 3:** Negative effects of smartphone use on teenagers.

Categorization (1st Level): Drawbacks		Σ
2nd Level	3rd Level	f (%) *
Health	Addiction/dependency	386 (22.65)
	Stress	216 (12.67)
	Insomnia	114 (6.69)
	Obesity	92 (5.39)
	Postural problems	89 (5.22)
	Visual impairment	76 (4.46)
Psychosocial	Sexting	284 (16.66)
	Nomophobia	252 (14.78)
	Phubbing	102 (5.98)
Academics	Low performance	268 (15.72)
	Decreased motivation	121 (7.10)
	Increased time wasting	93 (5.45)
Economics	Acquisition of other mobile devices	98 (5.75)
	Online shopping	59 (3.46)
	Gambling consumption	57 (3.34)
		Total: 1704

* Legend: Σ = summation; f = Absolute frequency; % = Relative frequency.

**Table 4:** News distribution by newspaper.

Digital Newspaper	Period: From 1 January 2018 to 31 December 2021												Σ
	J	F	M	A	M	J	J	A	S	O	N	D	f (%) *
*20minutos*					5	1	9		6	6	5	8	43 (2.52)
*ABC*	4	4	8	16	8	6	16	36	28	20	16	39	201 (11.7)
*alerta digital*							2						2 (0.11)
*Diario16*					3				4				7 (0.41)
*El Confidencial*	2							7	16				25 (1.46)
*EL CORREO*				14		2			8		6		30 (1.76)
*elDiario.es*		26		28	8	3	9	11	10	8	7	6	116 (6.80)
*EL ESPAÑOL*			5						6				11 (0.64)
*EL MUNDO*		5	8		6				17			14	50 (2.93)
*EL PAÍS*	8	7	11	12		6	5	6	11	6	8	12	92 (5.39)
*elPeriódico*	5								4				9 (0.52)
*Elplural*												8	8 (0.46)
*europa press*	8	18			8	7	21	9	32	22	16	21	162 (9.50)
*infoLibre*		6							3				9 (0.52)
*LA GACETA*	23			21	23				5				72 (4.22)
*LA VANGUARDIA*	28	32	17					17	41		39	7	181 (10.6)
*Público*			11							27			38 (2.23)
*Última hora*								21		44			65 (3.81)
*Diario de Sevilla*							6		4		3		13 (0.76)
*Aragón digital*												8	8 (0.46)
*HERALDO*			6							7	4		17 (0.99)
*EL DIARIO MONTAÑES*			7									1	8 (0.46)
*Lanza*							1		2				3 (0.17)
*EL ADELANTADO*	8	14			7	2		2					33 (1.93)
*El Norte de Castilla*		1				2			4			1	8 (0.46)
*LA GACETA DE SALAMANCA*	4		4	1	3	2	2	1	6		4	4	31 (1.81)
*La Nueva Crónica*		1	1							4	6		12 (0.70)
*Leonoticias*									8				8 (0.46)
*salamanca24h.com*	2				1			1	2		2	11	19 (1.11)
*ara.cat*		2			3								5 (0.29)
*Diari DE TARRAGONA*			1				4				1		6 (0.35)
*METROPOLI ABIERTA*									2				2 (0.11)
*getafe actualidad*										3			3 (0.17)
*INFORMACIÓN*									2		5	4	11 (0.64)
*Levante*			7		4								11 (0.64)
*EL DIARIO VASCO*											1		1 (0.05)
*Gasteizhoy*												2	2 (0.11)
*Noticias de Gipúzkoa*								1					1 (0.05)
*HOY*			1			2		2	1	1	1		8 (0.46)
*ElCorreoGallego*								1	16		3	1	21 (1.23)
*Faro de Vigo*						1	2		19	3		1	26 (1.52)
*La Región*								2	17				19 (1.11)
*La Voz de Galicia*		14		7	18	7	21	28	29	8		2	134 (7.86)
*La Opinión A CORUÑA*									8				8 (0.46)
*Diario de Mallorca*												2	2 (0.11)
*MENORCA.info*			4				3		2	5			14 (0.82)
*LA PROVINCIA*			2		1	2	1		2		2		10 (0.58)
*EL DÍA.es*											5	2	7 (0.41)
*DIARIO DE NAVARRA*												2	2 (0.11)
*EL COMERCIO*									3		2		5 (0.29)
*La Voz De Asturias*					1								1 (0.05)
*La Opinión De MURCIA*			1		2								3 (0.17)
*LA VERDAD*		6	11		8	6	8	15	19	10	20	18	121 (7.10)
News overall sum:	92	136	105	99	109	49	110	160	340	174	156	174	1704

* Legend: Σ = summation; f = Absolute frequency; % = Relative frequency.

**Table 5:** Distribution of news that attributes benefits to the use of the smartphone.

Subcategories (2nd Level)		Skills Development	Tool Availability	Development of Feelings/Emotions	Academic Opportunities	Σ
						f (%) *
Number of news items per month (period: from 1 January 2018 to 31 December 2021)	January	25	5	1	2	33 (1.93)
	February	30	11	2	17	70 (4.10)
	March	58	35	16	3	112 (6.57)
	April	39	38	9	8	94 (5.51)
	May	78	19	6	2	105 (6.16)
	June	62	26	4	9	101 (5.92)
	July	80	14	9	3	106 (6.22)
	August	47	11	3	41	102 (5.98)
	September	197	164	93	81	535 (31.39)
	October	62	71	62	36	231 (13.55)
	November	94	112	86	29	321 (18.83)
	December	186	192	112	78	568 (33.33)
	Total:	958	698	413	309	

* Legend: Σ = summation; f = Absolute frequency; % = Relative frequency.

**Table 6:** Distribution of news that attribute inconveniences to the use of the smartphone.

Subcategories (2nd Level)		Health	Psychosocial	Academics	Economics	Σ
						f (%) *
Number of news items per month (period: from 1 January 2018 to 31 December 2021)	January	68	46	2	5	119 (6.98)
	February	24	84	3	13	124 (7.27)
	March	90	68	21	3	179 (10.50)
	April	95	64	2	3	144 (8.45)
	May	45	37	4	24	113 (6.63)
	June	102	27	80	25	238 (13.96)
	July	87	48	80	63	278 (16.31)
	August	96	36	75	6	213 (12.5)
	September	123	96	67	38	324 (19.01)
	October	62	19	57	12	150 (8.80)
	November	47	42	46	9	144 (8.45)
	December	134	89	45	13	281 (16.49)
	Total:	973	638	482	214	

* Legend: Σ = summation; f = Absolute frequency; % = Relative frequency.

## Data Availability

Not applicable.
